# The Methoxyflavonoid Isosakuranetin Suppresses UV-B-Induced Matrix Metalloproteinase-1 Expression and Collagen Degradation Relevant for Skin Photoaging

**DOI:** 10.3390/ijms17091449

**Published:** 2016-09-01

**Authors:** Hana Jung, Eunjoo H. Lee, Tae Hoon Lee, Man-Ho Cho

**Affiliations:** 1 Graduate School of East-West Medical Sciences, Kyung Hee University, Yongin 17104, Korea; sbsbc@khu.ac.kr (H.J.); ehwang@khu.ac.kr (E.H.L.); 2 Graduate School of Biotechnology, Kyung Hee University, Yongin 17104, Korea

**Keywords:** isosakuranetin, anti-photoaging, methoxyflavonoid, MMP-1, UV-B-radiation

## Abstract

Solar ultraviolet (UV) radiation is a main extrinsic factor for skin aging. Chronic exposure of the skin to UV radiation causes the induction of matrix metalloproteinases (MMPs), such as MMP-1, and consequently results in alterations of the extracellular matrix (ECM) and skin photoaging. Flavonoids are considered as potent anti-photoaging agents due to their UV-absorbing and antioxidant properties and inhibitory activity against UV-mediated MMP induction. To identify anti-photoaging agents, in the present study we examined the preventative effect of methoxyflavonoids, such as sakuranetin, isosakuranetin, homoeriodictyol, genkwanin, chrysoeriol and syringetin, on UV-B-induced skin photo-damage. Of the examined methoxyflavonoids, pretreatment with isosakuranetin strongly suppressed the UV-B-mediated induction of MMP-1 in human keratinocytes in a concentration-dependent manner. Isosakuranetin inhibited UV-B-induced phosphorylation of mitogen-activated protein kinase (MAPK) signaling components, ERK1/2, JNK1/2 and p38 proteins. This result suggests that the ERK1/2 kinase pathways likely contribute to the inhibitory effects of isosakuranetin on UV-induced MMP-1 production in human keratinocytes. Isosakuranetin also prevented UV-B-induced degradation of type-1 collagen in human dermal fibroblast cells. Taken together, our findings suggest that isosakuranetin has the potential for development as a protective agent for skin photoaging through the inhibition of UV-induced MMP-1 production and collagen degradation.

## 1. Introduction

Skin aging can be characterized by two main processes, intrinsic and extrinsic aging. The impairment of skin integrity is one of the most prominent indications of skin aging, which occurs as a consequence of exposure to environmental factors [1]. Extrinsic aging is generally referred to as photoaging and is characterized by wrinkling and pigmentary changes of the skin. Solar ultraviolet (UV) radiation, particularly UV-A (315–400 nm) and UV-B radiation (280–315 nm), is one of the most important extrinsic aging factors [2]. UV-A composing more than 90% of solar UV radiation can penetrate deep into the dermis and causes structural damage to the DNA, impairment of the immune system and skin cancer [3,4]. Although UV-B makes up 4% to 5%, it is the most active component of solar UV radiation [2,3,4]. Chronic exposure of the skin to UV-B radiation results in activation of pro-inflammatory cytokines and inflammatory mediators, stimulation of the mitogen-activated protein kinase (MAPK) signaling pathway, and increased production of matrix metalloproteinases (MMPs), which consequently cause alterations in the extracellular matrix (ECM) and accelerate skin aging [5,6,7]. MMPs are released from keratinocytes and dermal fibroblasts by solar stress and participate in proteolysis of the ECM. In particular, MMP-1, also known as interstitial collagenase, is mainly responsible for the degradation of dermal type 1 collagen that is the most abundant structural protein in the dermis. Collagen degradation and inhibition of collagen synthesis deteriorate the structural integrity of the skin during photoaging [8,9,10,11]. Natural products possessing the ability to promote collagen synthesis or inhibit the major collagen-degradation enzymes are, therefore, possible to use as anti-photoaging cosmetic and therapeutic agents.

Natural phenolic compounds, including flavonoids, have been thought to be potent beneficial agents for skin photoaging due to their UV-absorbing and antioxidant properties [3,4]. They are also known to be involved in the suppression of the UV-induced signaling cascade for MMP expression during skin photoaging [3,4,12,13,14]. Flavonoids, such as quercetin, apigenin and luteolin, were reported to have preventing effects on skin photo-damage [3,12,13,14]. Quercetin inhibits MMP-1 expression in human dermal fibroblasts treated with UV-A and 12-*O*-tetradecanoylphobol 13-acetate (TPA) through the suppression of MAP kinase and activator protein-1-dependent signaling pathways [12,13]. Apigenin and luteolin also reported to inhibit UVA-induced MMP-1 expression in human keratinocytes [14].

Flavonoids often exist as methylated forms, which have different biological activities compared to non-methylated flavonoids [15,16]. The polymethoxyflavone nobiletin was reported to prevent UV-B-induced photo-inflammation and photoaging in human keratinocytes [17]. The 8-methoxyflavone wogonin inhibits the TPA-induced MMP-1 expression in human dermal fibroblasts [13]. These findings suggested that methoxyflavonoids are potent beneficial agents for skin photoaging. In this regard, we scrutinized the preventative activity of various naturally occurring methoxyflavonoids, such as sakuranetin, isosakuranetin, homoeriodictyol, genkwanin, chrysoeriol and syringetin, to skin photoaging. Of the examined methoxyflavonoids, isosakuranetin inhibited UV-B-induced MMP-1 expression and collagen degradation in human keratinocyte HaCaT cells and human dermal fibroblasts (NHDF cells).

## 2. Results and Discussion

### 2.1. Effect of Methoxyflavonoids on Ultraviolet (UV)-B-Induced Matrix Metalloproteinase-1 (MMP-1) Expression in Human Keratinocytes

**Figure 1:**
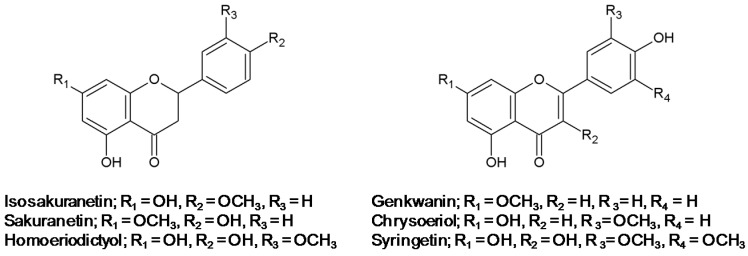
Structure of methoxyflavonoids examined in this study.

Collagen is the major structural component of the ECM of dermal connective tissue and its reduction has been suggested to cause skin photoaging through acceleration of skin wrinkle formation and impairment of the skin integrity [10]. MMP-1 is induced in response to UV radiation and is mainly responsible for collagen degradation in dermal tissues during photoaging [8,9,10,11]. In the present study, the effects of methoxyflavonoids on the UV-induced MMP-1 expression and collagen degradation in human dermal cells were investigated to identify potent anti-photoaging agents. The methoxyflavonoids with different flavonoid backbones used were mono-methoxyflavanones (sakuranetin, isosakuranetin and homoeriodictyol), mono-methoxyflavones (genkwanin and chrysoeriol) and a di-methoxyflavonol (syringetin) (Figure 1).

**Figure 2:**
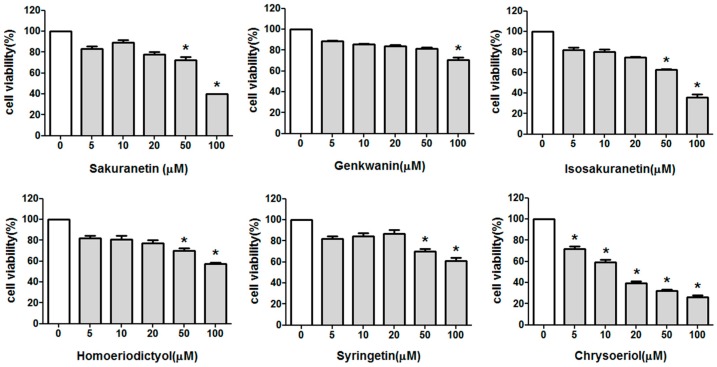
Effect of methoxyflavonoids on the cell viability by MTT assay. HaCaT cells were grown in the culture medium in the presence of various concentrations of methoxyflavonoids for 24 h. Cell viability was measured by MTT assays. All data are given as means ± SD of at least three independent experiments with triplicate samples. * *p* ˂ 0.05 vs. medium alone.

We first examined the effects of methoxyflavonoids on the cell viability by the colorimetric 3-(4,5-dimetnythiazol-2-yl)-2,5-diphenyl-thetazolium bromide (MTT) assay. The results showed that sakuranetin, genkwanin, isosakuranetin, homoeriodictyol and syringetin have no effect on the cell viability up to 20 μM of each compound. In the case of isosakuranetin, cell viability was about 64% at the concentration of 50 μM during 24 h treatment (Figure 2), while chrysoeriol resulted in HaCaT cell death by about 74% at 50 μM compared to that of untreated cells in a concentration-dependent manner. These results indicate that all methoxyflavonoids except for chrysoeriol have no significant cytotoxicity at the concentration used in the present study.

**Figure 3:**
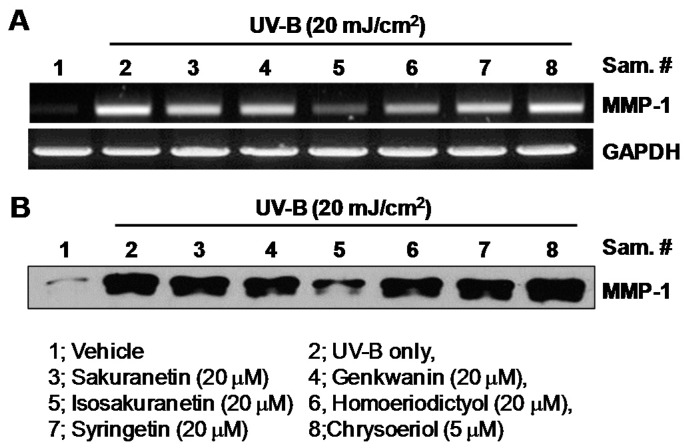
Effect of methoxyflavonoids on matrix metalloproteinase-1 (MMP-1) expression in ultraviolet (UV)-B-irradiated HaCaT cells. (**A**) HaCaT cells were pretreated with methoxyflavonoids for 24 h and then irradiated with UV-B (20 mJ/cm^2^). UV-irradiated cells were then cultured for another 24 h. Levels of MMP-1 mRNA were evaluated by RT-PCR. Glyceraldehyde-3-phosphate dehydrogenase (GAPDH) mRNA was used as internal control; (**B**) Levels of MMP-1 proteins were measured by Western blot analysis using a monoclonal antibody against human MMP-1.

The effect of methoxyflavonoids on MMP-1 expression in the human keratinocyte HaCaT cells was examined to screen potent agents for anti-photoaging. MMP-1 mRNA levels in UV-B-irradiated HaCaT cells were determined in the presence of sakuranetin, genkwanin, isosakuranetin, homoeriodictyol and syringetin at a 20 μM concentration. In the case of chrysoeriol, MMP-1 mRNA levels were determined at a 5 μM concentration because of its cytotoxicity to HaCaT cells (Figure 2). Upon stimulation by UV-B irradiation, the levels of the MMP-1 transcript and protein increased markedly in HaCaT cells. RT-PCR analysis showed that the treatment of isosakuranetin strongly inhibits the UV-induced MMP-1 mRNA expression in HaCaT cells (Figure 3A). The other methoxyflavonoids showed no significant inhibition effect on the UV-B-induced MMP-1 mRNA expression (Figure 3A). In parallel with the mRNA level, isosakuranetin remarkably inhibited the induction of the MMP-1 protein in UV-B-irradiated HaCaT cells, whereas the other methoxyflavonoids showed almost no effect on the MMP-1 protein level (Figure 3B).

**Figure 4:**
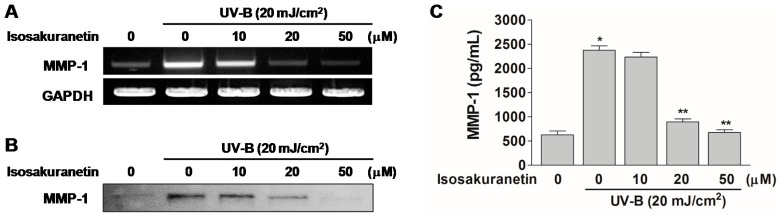
Effect of isosakuranetin on MMP-1 expression in UV-B-irradiated HaCaT cells. HaCaT cells were pretreated with various concentration of isosakuranetin for 24 h and then irradiated with UV-B (20 mJ/cm^2^) for 15 s. UV-irradiated cells were then cultured for another 24 h. Levels of MMP-1 mRNA were evaluated by RT-PCR. GAPDH mRNA was used as internal control (**A**); Levels of MMP-1 proteins were measured by Western blot analysis using a monoclonal antibody against human MMP-1 (**B**) and quantified by enzyme-linked immunosorbent assay (ELISA) (**C**). All data are given as means ± SD of at least three independent experiments with triplicate samples. * *p* < 0.05 vs. negative control, ** *p* < 0.05 vs. positive control.

To confirm its inhibitory effect on the UV-B-induced MMP-1 expression, HaCaT cells were pretreated with different concentrations (10, 20 and 50 μM) of isosakuranetin and the MMP-1 transcript and protein levels were analyzed. The result showed that the UV-B-mediated induction of the MMP-1 mRNA and protein was inhibited by pretreatment with isosakuranetin in a concentration-dependent manner (Figure 4).

The effect of isosakuranetin on the MMP-1 proteins in UV-B-irradiated HaCaT cells was quantitatively analyzed by enzyme-linked immunosorbent assay (ELISA). In the non-treated cells, the production of MMP-1 increased up to 2440.1 ± 173.53 pg/mL in response to UV irradiation, compared to the basal levels of 725.66 ± 74.63 pg/mL. Meanwhile, isosakuranetin dose-dependently inhibited the UV-B-induced production of MMP-1 proteins. The amount of the MMP-1 proteins in UV-B-irradiated HaCaT cells was reduced to 895.37 ± 62.12 and 679.19 ± 54.23 pg/mL by pretreatment with isosakuranetin at 20 and 50 μM concentrations, respectively (Figure 4C). It was previously reported that the pretreatment of 5 mM of apigenin and luteolin inhibits the UV-A-induced MMP-1 expression in HaCaT cells by about 60% and 70%, respectively [14]. Quercetin and luteolin were reported to inhibit the UV-A-induced MMP-1 expression in human dermal fibroblasts by about 60% and 90% at a 10 μM concentration of pretreatment, respectively [12]. Our result showed that pretreatment with 20 μM of isosakuranetin suppressed the UV-B-induced MMP-1 expression in HaCaT cells by 90% (Figure 4C), suggesting that it is a potent protective agent for the UV-mediated skin photo-damage.

### 2.2. Isosakuranetin Inhibits UV-B-Induced MMP-1 Expression through the Suppression of ERK1/2 Kinase Pathways

**Figure 5:**
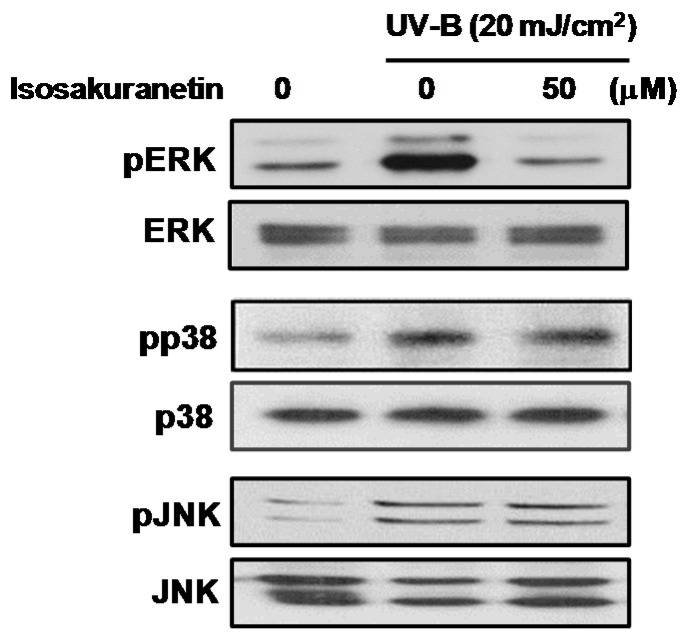
Effect of isosakuranetin on UV-B-induced phosphorylation of MAPKs in HaCaT cells. HaCaT cells were pretreated with indicated concentrations of isosakuranetin for 24 h and then irradiated with UV-B (20 mJ/cm^2^), and then cells were further cultured for 1 h. Phosphorylation of ERK, JNK, and p38 MAP kinases was determined by specific antibodies against the phosphorylated form of MAP kinases.

It is well known that MAPK signaling pathways play an important role in MMP-1 expression during UV-induced skin photoaging [5,6]. Previously identified anti-photoaging flavonoids, including quercetin, apigenin and luteolin, were reported to inhibit the phosphorylation of the MAPK signaling components such as ERK, p38 and JNK [13,14]. To elucidate the molecular basis of the inhibitory effects of isosakuranetin on the expression of MMP-1 mRNA and protein, we examined the levels of UV-B-induced phosphorylation of MAP kinases in HaCaT cells treated with isosakuranetin. The exposure of HaCaT cells to UV-B irradiation resulted in a remarkable increase of phosphorylated MAPK signaling components, such as ERK1/2, JNK1/2 and p38 proteins. The results showed that isosakuranetin significantly inhibited UV-induced phosphorylation of ERK1/2 MAPKs. In contrast, isosakuranetin barely affected the phosphorylation of p38 and JNK (Figure 5). These results suggest that the ERK1/2 kinase pathways may contribute to the inhibitory effects of isosakuranetin on the production of MMP-1 in UV-irradiated HaCaT cells.

### 2.3. Isosakuranetin Protects Collagen Degradation from UV-B Radiation

**Figure 6:**
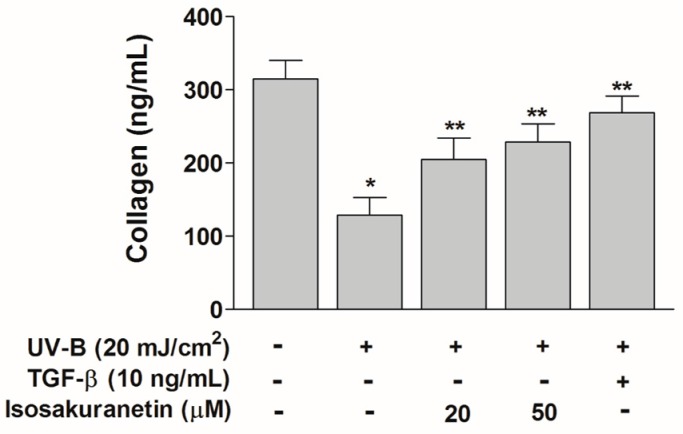
Effect of isosakuranetin on the level of type-1 procollagen in UV-B-irradiated NHDF cells. After pretreatment of fibroblast cells with different concentrations of isosakuranetin for 24 h, cells were irradiated with UV-B (20 mJ/cm^2^), and then cells were further incubated for 24 h. Total collagen levels were measured in the culture media of control cells or UV-B-treated cells by SirCol assay. Data shown are mean values ± S.D. * *p* < 0.05 vs. non-UV-B-irradiated cells, ** *p* < 0.05 vs. UV-B-irradiated cells.

Exposure to UV-B radiation reduces type-1 procollagen levels in human skin [10]. In order to investigate the effects of isosakuranetin on the degradation of type-1 procollagen, we examined the protein levels of procollagen in UV-B-irradiated human fibroblasts (NHDF cells) in the presence or absence of isosakuranetin by SirCol collagen assay. Upon UV-B irradiation, procollagen was degraded to basal levels in NHDF cells, whereas it was decreased to a lesser extent in isosakuranetin-treated cells (Figure 6). It has been reported that UV irradiation suppresses the transforming growth factor β (TGF-β)/smad signaling pathway, which regulates the cellular matrix synthesis and tissue generation process through the production of type-1 collagen [18]. Therefore, we examined the effect of TGF-β on collagen degradation in the UV-B-irradiated NHDF cells. The collagen degradation was mostly protected by TGF-β treatment in the UV-B-irradiated NHDF cells (Figure 6). In UV-B-irradiated NHDF cells, the collagen levels in the isosakuranetin-treated cells were comparable with that in TGF-β-treated cells. This result suggested that isosakuranetin can protect ECM integrity of the dermal tissue from harmful UV-B radiation through the inhibition of collagen degradation and possibly by TGF-β-dependent enhanced collagen synthesis.

## 3. Materials and Methods

### 3.1. Cell Culture and Reagents

The human keratinocyte HaCaT cells were grown in Dulbecco’s modified Eagle’s medium (DMEM) supplemented with 10% Fetal Bovine Serum (FBS) and antibiotics (100 unit/mL penicillin, 100 µg/mL streptomycin) at 37 °C in a humidified incubator containing 5% CO_2_ and 95% air. DMEM, FBS, antibiotics, and trypsin EDTA was obtained from Invitrogen (Carlsbad, CA, USA). Primary cultures of dermal fibroblast were established from human foreskins. NHDF were maintained and grown in FGM-2 medium (Clonetics, Walkersville, MD, USA) containing 2% FBS, 0.1% recombinant human fibroblast growth factor (rhFGF) (Invitrogen), 0.1% insulin, 5 units/mL heparin, 100 units/mL of penicillin and 100 µg/mL streptomycin in a humidified incubator containing 5% CO_2_ at 37 °C. Specific antibodies against phosphor-ERK, total ERK, phosphor-p38, total p38, phosphor-JNK and total JNK were obtained from Santa Cruz biotechnology (Santa Cruz, CA, USA). Antibodies against human MMP-1 and human procollagen were obtained from R&D Systems (Minneapolis, MN, USA). Horse radish peroxidase (HRP)-conjugated anti-mouse and anti-rabbit IgG antibodies were obtained from Sigma-Aldrich (St. Louis, MO, USA). Other chemicals were obtained commercially from Sigma-Aldrich. Methoxyflavonoids, isosakuranetin, homoeriodictyol, chrysoeriol and syringetin, were purchased from Indofine Chemical Company (Hillsborough, NJ, USA). Genkwanin was bought from Extrasynthese (Genay Cedex, France). Sakuranetin was previously obtained from UV-irradiated rice leaves [19,20].

### 3.2. Cell Viability Assay

Cells were seeded in 96-well culture plates at 5 × 10^4^ cells/well and allowed to attach for 18 h. After discarding the growth medium, HaCaT cells were treated with indicated concentrations of each flavonoid compound in serum-free medium for 24 h. To examine cell viability, MTT (50 µg/mL) was added to each well and followed by 2 h incubation. MTT assay is the most common colorimetric method for cytotoxicity analysis, which quantifies the mitochondrial succinate dehydrogenase enzyme activity in metabolically active cells [21]. The cell-free supernatants were obtained from each well and 100 µL of dimethyl sulfoxide was added to the supernatants. Absorbance at 540 nm was measured using the spectrophotometric microplate reader Multiskan MS (Thermo Electron Corp., Waltham, MA, USA).

### 3.3. UV-B Irradiation

HaCaT and NHDF cells were incubated with or without each flavonoid compound for 24 h in serum free media. After pretreatments, cells were rinsed twice with phosphate-buffered saline (PBS) and followed by UV-B (20 mJ/cm^2^) irradiation at 312 nm using the UV Bio-Sun Lamps (Vilber Lourmat, Marine, France). UV-B radiation was uniformly irradiated to samples at a distance of 15 cm for 15 s with monitoring the radiation intensities using the UV-photodetector JIC 119 (BEC, Brookline, MA, USA) [22]. After irradiation, cells were added with or without flavonoids in serum free media and followed by additional 24 h incubation. Non-flavonoid treated controls with or without UV-B treatments were prepared by the same schedule of medium changes described above.

### 3.4. Reverse Transcriptase-Polymerase Chain Reaction (RT-PCR)

Total RNAs were prepared from HaCaT cells with or without treatment of UV and each flavonoid compound using a Trizol Reagent kit (Invitrogen) as previously described [23]. First-strand cDNAs were generated with total RNA (2 µg) as templates using the M-MuLV reverse transcriptase (Fermentas Life Science, Glen Burnie, MD, USA). The primer sets for PCR amplification were as follows: MMP-1, forward 5′-AGCGTGTGACAGTAAGCTAA-3′ and reverse 5′-GTTTTCCTCAGAAAGAGCAGCAT-3′; GAPDH mRNA levels were used as internal controls.

### 3.5. Western Blot Analysis

Cells were collected and resuspended in radio immunoprecipitation assay buffer (150 mM sodium chloride, 1% Triton X-100, 1% sodium deoxycholate, 0.1% sodium dodecyl sulfate (SDS), 50 mM Tris–HCl, pH 7.5, 2 mM ethylenediaminetetraacetic acid) containing proteinase inhibitor cocktails (Roche, Indianapolis, IN, USA). Concentrations of total proteins were determined with a protein assay kit (Bio-Rad Laboratories, Philadelphia, PA, USA). Protein samples (30 µg/lane) were resolved with 8%–12% SDS-polyacrylamide gel, and transferred to nitrocellulose membrane. Target proteins were detected by Western blot as described previously [24]. HRP-conjugated secondary antibodies were used in Western blot analysis. Western signals were developed with an enhanced chemiluminescence system from GE healthcare (Buckinghamshire, UK) and exposed to X-ray film (Fuji Photo Film Co., Ltd., Tokyo, Japan).

### 3.6. Enzyme-Linked Immunosorbent Assay

HaCaT cells were pre-incubated in six-well plates in the presence or absence of isosakuranetin for 18 h and then irradiated with UV-B radiation. MMP-1 protein in culture supernatants were analyzed by ELISA kits (R&D Systems) according to the manufacturer’s instructions.

### 3.7. Measurement of Procollagen

To determine total soluble collagen in culture supernatants, the SirCol collagen assay kit (Biocolor Ltd., Belfast, Northern Ireland) was used. Flavonoid compounds were added to confluent fibroblast cells in 24-well plates and the cultures were incubated for 24 h. After UV irradiation as described above, cell-free supernatants were obtained from the culture, and Sirius red dye (1 mL) was added to the 400 µL of supernatant followed by incubation for 30 min at room temperature with gentle agitation. The collagen-bound dye precipitates were harvested by centrifugation at 12,000× *g* for 10 min. The resulting collagen-bound dye was redissolved with 1 ml of 0.5 M NaOH, and absorbance at 540 nm was measured using a microplate ELISA reader (Dynex Technologies, Chantilly, VA, USA).

### 3.8. Statistical Analysis

In the present study, all experiments were performed with triplicate samples and repeated at least three times. Means ± SD of the data were presented in this study, and statistical significance between groups was determined using 1-way ANOVA analysis followed by Student’s *t*-test.

## 4. Conclusions

In conclusion, we examined the protective effect of diverse methoxyflavonoids on skin markers of photoaging using an in vitro model system and found that isosakuranetin inhibited MMP-1 expression and collagen degradation in UV-B-irradiated HaCaT and NHDF cells. Our results showed that isosakuranetin inhibits UV-B-induced MMP-1 expression in HaCaT cells through the inhibition of UV-B-induced phosphorylation of ERK1/2. These findings suggest that isosakuranetin is a potent protective agent suppressing the UV-B-induced MMP-1 expression and collagen degradation relevant for photoaging.

Although isosakuranetin showed inhibitory effects on UV-B-mediated MMP-1 expression and collagen degradation, it could have different effects on these skin markers of photo-damage in epidermal cells irradiated with different light sources, such as solar-simulated and UV-A radiations. Therefore, the effects of isosakuranetin on markers of skin photo-damage in epidermal cells irradiated with different light sources were further examined to elucidate its precise protective role on skin photoaging.

## References

[1] Farage M.A., Miller K.W., Elsner P., Maibach H.I. (2008). Intrinsic and extrinsic factors in skin ageing: A review. Int. J. Cosmet. Sci..

[2] Jenkins G. (2002). Molecular mechanisms of skin ageing. Mech. Ageing Dev..

[3] Svobodová A., Psotová J., Walterová D. (2003). Natural phenolics in the prevention of UV-induced skin damage. A review. Biomed. Pap..

[4] Afaq F., Mukhtar H. (2006). Botanical antioxidants in the prevention of photocarcinogenesis and photoaging. Exp. Dermatol..

[5] Im A.R., Nam K.W., Hyun J.W., Chae S. (2016). Phloroglucinol reduces photodamage in hairless mice via matrix metalloproteinase activity through MAPK pathway. Photochem. Photobiol..

[6] Chen C.C., Chiang A.N., Liu H.N., Chang Y.T. (2014). EGb-761 prevents ultraviolet B-induced photoaging via inactivation of mitogen-activated protein kinases and proinflammatory cytokine expression. J. Dermatol. Sci..

[7] Kim M.S., Oh G.H., Kim M.J., Hwang J.K. (2013). Fucosterol inhibits matrix metalloproteinase expression and promotes type-1 procollagen production in UVB-induced HaCaT cells. Photochem. Photobiol..

[8] McCawley L.J., Matrisian L.M. (2001). Matrix metalloproteinases: They’re not just for matrix anymore!. Curr. Opin. Cell Biol..

[9] Lauer-Fields J.L., Juska D., Fields G.B. (2002). Matrix metalloproteinases and collagen catabolism. Biopolymers.

[10] Brennan M., Bhatti H., Nerusu K.C., Bhagavathula N., Kang S., Fisher G.J. (2003). Matrix metalloproteinase-1 is the major collagenolytic enzyme responsible for collagen damage in UV-irradiated human skin. Photochem. Photobiol..

[11] Visse R., Nagase H. (2003). Matrix metalloproteinases and tissue inhibitors of metalloproteinases. Structure, function, and biochemistry. Circ. Res..

[12] Sim G.S., Lee B.C., Cho H.S., Lee J.W., Kim J.H., Lee D.H., Kim J.H., Pyo H.B., Moon D.C., Oh K.W. (2007). Structure activity relationship of antioxidative property of flavonoids and inhibitory effect on matrix metalloproteinase activity in UVA-irradiated human dermal fibroblast. Arch. Pharm. Res..

[13] Lim H., Kim H.P. (2007). Inhibition of mammalian collagenase, matrix metalloproteinase-1, by naturally-occurring flavonoids. Planta Med..

[14] Hwang Y.P., Oh K.N., Yun H.J., Jeong H.G. (2011). The flavonoids apigenin and luteolin suppress ultraviolet A-induced matrix metalloproteinase-1 expression via MAPKs and AP-1-dependent signaling in HaCaT cells. J. Dermatol. Sci..

[15] Aida Y., Tamogami S., Kodama O., Tsukiboshi T. (1996). Synthesis of 7-methoxyapigeninidin and its fungicidal activity against *Gloeocercospora sorghi*. Biosci. Biotechnol. Biochem..

[16] Zhang L., Kong Y., Wu D., Zhang H., Wu J., Chen J., Ding J., Hu L., Jiang H., Shen X. (2008). Three flavonoids targeting the β-hydroxyacyl-acyl carrier protein dehydratase from *Helicobacter pylori*: Crystal structure characterization with enzymatic inhibition assay. Protein Sci..

[17] Tanaka S., Sato T., Akimoto N., Yano M., Ito A. (2004). Prevention of UVB-induced photoinflammation and photoaging by a polymethoxy flavonoid, nobiletin, in human keratinocytes in vivo and in vitro. Biochem. Pharmacol..

[18] Quan T., He T., Kang S., Voorhees J.J., Fisher G.J. (2002). Ultraviolet irradiation alters transforming growth factor β/smad pathway in human skin in vivo. J. Investig. Dermatol..

[19] Park J.H., Fu Y.Y., Chung I.S., Hahn T.R., Cho M.H. (2013). Cytotoxic property of UV-induced rice phytoalexins to human colon carcinoma HCT-116 Cells. J. Korean Soc. Appl. Biol. Chem..

[20] Park H.L., Lee S.W., Jung K.H., Hahn T.R., Cho M.H. (2013). Transcriptomic analysis of UV-treated rice leaves reveals UV-induced phytoalexin biosynthetic pathways and their regulatory networks in rice. Phytochemistry.

[21] Berridge M.V., Tan A.S. (1993). Characterization of the cellular reduction of 3-(4,5-dimethylthiazol-2-yl)-2,5-diphenyltetrazolium bromide (MTT): Subcellular localization, substrate dependence, and involvement of mitochondrial electron transport in MTT reduction. Arch. Biochem. Biophys..

[22] Park M., Han J., Lee C.S., Soo B.H., Lim K.M., Ha H. (2013). Carnosic acid, a phenolic diterpene from rosemary, prevents UV-induced expression of matrix metalloproteinases in human skin fibroblasts and keratinocytes. Exp. Dermatol..

[23] Lee T.H., Kwak H.B., Kim H.H., Lee Z.H., Chung D.K., Baek N.I., Kim J. (2007). Methanol extracts of Stewartia koreana inhibit cyclooxygenase-2 (COX-2) and inducible nitric oxide synthase (iNOS) gene expression by blocking NF-κB transactivation in LPS-activated RAW 264.7 cells. Mol. Cells.

[24] Baumann B., Weber C.K., Troppmair J., Whiteside S., Israel A., Rapp U.R., Wirth T. (2000). Raf induces NF-κB by membrane shuttle kinase MEKK1, a signaling pathway critical for transformation. Proc. Natl. Acad. Sci. USA.

